# Risk factors for periappendiceal adhesions in acute appendicitis: a retrospective comparative study

**DOI:** 10.1186/s12893-022-01579-y

**Published:** 2022-04-08

**Authors:** Shenshuo Gao, Xiaobo Guo, Leping Li, Changqing Jing, Yan Ma

**Affiliations:** 1 Department of Gastroenterological Surgery, Shandong Provincial Hospital, Cheeloo College of Medicine, Shandong University, Jinan, 250021 Shandong China; 2 Department of Gastroenterological Surgery, Shandong Provincial Hospital Affiliated to Shandong First Medical University, Jinan, 250021 Shandong China

**Keywords:** Appendectomy, Adhesions, Risk factors, Perforation, Suppuration

## Abstract

**Purpose:**

Acute appendicitis usually requires immediate surgical treatment, but appendectomies were difficult for some patients with severe periappendiceal adhesions. We investigated risk factors of intraoperative adhesions to help surgeons make better treatment plans for appendicitis.

**Methods:**

We retrospectively analyzed 186 cases diagnosed with acute appendicitis and underwent surgery in Shandong Provincial Hospital affiliated to Shandong First Medical University between January 2018 and December 2019. According to the degree of intraoperative adhesions, they were divided into mild, moderate and severe groups. Then, we analyzed a number of preoperative factors contributed to adhesions, suppuration and perforation during appendectomy in 186 patients.

**Results:**

Contrast to the moderate group (MoG) and the mild group (MiG), the severe degree of adhesions group (SG) had a higher intraoperative perforation and suppuration rate, a greater likelihood of conversion to open and more postoperative complications. Multivariable logistic regression analysis showed that recurrent appendicitis and high neutrophil percentage were independently associated with periappendiceal adhesions. The preoperative ultrasonography (US) revealed periappendiceal fluid and high neutrophil percentage were independently associated with appendix suppuration. A high preoperative neutrophil percentage was independently associated with appendix perforation.

**Conclusions:**

Recurrent appendicitis and preoperative high neutrophil percentage were risk factors of periappendiceal adhesions; preoperative US revealed periappendiceal fluid and high neutrophil percentage were risk factors of appendix suppuration; and a high preoperative neutrophil percentage was a risk factor of appendix perforation.

## Introduction

Appendicitis is a global disease. The incidence of appendicitis is stable in most Western countries. But data from newly industrialized countries suggests that appendicitis is rising rapidly [1]. Appendicitis has a high incidence and is one of the most common diseases in abdominal emergency. Although some studies suggested that antibiotic treatment can cure acute appendicitis or can be the first line of treatment [2–5], appendectomy is still the main surgical method for the treatment of appendicitis. Current evidence shows laparoscopic appendectomy (LA) was the most effective surgical treatment, being associated with a lower incidence of wound infection, lower pain intensity on day one, shorter hospital stay, earlier food tolerance, earlier return to work and better quality of life scores when compared to open appendectomy (OA) [6–9]. Though the majority of patients with acute appendicitis can be successfully managed with laparoscopy, some operations were initiated laparoscopically but were converted to the open approach because of technical limitations, body habitus, prior surgery, more advanced disease, or surgical inexperience [10, 11]. The conversion to open rate during laparoscopic appendectomy is ~ 4% [12]. It had a higher likelihood of complications compared to OA [10]. The most common reason for open conversion were severe acute inflammation and adhesions [13]. Obviously, severe acute inflammation and adhesions can make appendectomy more difficult and even cause more complications. The aim of our study is to evaluate preoperative risk factors that are contributed to adhesions and suppuration in LA and OA, and to help surgeons make better decision before operations.

## Methods

We retrospectively analyzed 186 cases diagnosed with acute appendicitis and underwent surgery in Shandong Provincial Hospital affiliated to Shandong First Medical University between January 2018 and December 2019, including 144 cases with LA and 42 cases with OA. Exclusion criteria include: non-operative treatment cases, cases with previous history of major abdominal surgery, cases with unclear intraoperative adhesions degree, cases with postoperative pathological confirmation of appendiceal tumor, and cases younger than 14 years old. All the data were obtained from the patients’ medical records, and the study was approved by the ethics committee of the study center.

This study collected and analyzed the information of patients including age, gender, whether the first onset of appendicitis, time interval from symptom onset to operation, time interval from initial symptom onset to operation of recurrent cases, preoperative leukocyte count and neutrophil percentage within 24 h, whether preoperative ultrasonography(US) revealed periappendiceal fluid, the operation time, the degree of periappendiceal adhesions, appendix situation of perforation, appendix situation of suppuration, whether extended resection, whether conversion to open surgery, postoperative complications, total length of hospital stay.

The patients who were not clearly diagnosed with appendicitis before this hospitalization were the first onset, and the others were recurrent. For all surgical patients, time interval from symptom onset to operation was divided into four groups: ≤ 1 day (group A), > 1 day and ≤ 2 days (group B), > 2 days and ≤ 3 days (group C), and > 3 days (group D). For recurrent patients, time interval from initial symptom onset to operation was divided into three groups: ≤ 3 months (group E), > 3 months and ≤ 12 months (group F), and > 12 months (group G). Whether perforation, suppuration or convert to open during surgery is according to medical records. Extended resection including partial cecectomy, right hemicolectomy or partial small bowel resection. Postoperative complications mainly include within 30 days of postoperative incision infection, incision hernia, intestinal obstruction, abdominal abscess, pulmonary infection, lower limb vein thrombosis and so on.

According to the description of the surgical records, the appendiceal adhesion extent was classified into three degrees [14]: mild (no obvious adhesion or light adhesions, which can easily be separated by blunt dissection), moderate (adhesions where blunt dissection is possible but sharp dissection necessary, with vascularization), and severe (lysis of the adhesions is possible by sharp dissection only, organs are strongly attached, and organ damage is hard to prevent). Intraoperative details and early postoperative outcomes were compared between three groups, and Univariate and multivariate analyses were used to study the risk factors of intraoperative adhesions, suppuration, and perforation.

### Statistical analysis

All analyses were performed using IBM SPSS Statistics for Windows version 25 (IBM Corp., Armonk, NY, USA). Mann–Whitney U test or Kruskal–Wallis H test were used for non-normally distributed continuous variables, which were shown as the median and range. Categorical variables were analyzed using the χ^2^ test or Fisher’s exact test. Multivariable logistic regression was performed to identify independent risk factors associated with intraoperative adhesions, suppuration and perforation during appendectomy, and the results expressed as odds ratios (ORs) and 95% confidence intervals (95% CIs). *P* values of < 0.05 were considered statistically significant.

## Results

### Patients’ demographics and clinical characteristics

Among the 186 cases of appendectomy patients included in the analysis, 89 were male and 97 were female, 118 were the first onset and 68 were recurrent, 144 patients underwent LA and 42 patients underwent OA. Groups of time interval from symptom onset to operation are as follows: group A 64, group B 58, group C 22, group D 36, and 6 patients that onset time were not clearly record. Recurrent groups of time interval from initial symptom onset to operation are as follows: group E 9, group F 25, group G 29, and 5 patients that onset time were not clearly record. 35 cases were found to have periappendiceal fluid by preoperative US examinations, 147 cases were not, and 4 cases had no preoperative US examination. There were 103 cases of suppuration and 83 cases of non-suppuration. There were 92 cases with mild, 34 cases with moderate and 60 cases with severe abdominal adhesions. 27 cases were found to have perforated appendix. The average operation time was 76 min. The preoperative WBC count was 11.4 (range from 2.1 to 24.3) and the preoperative neutrophil percentage was 74.6 (range from 30.5 to 95.8). 115 cases were placed a drainage tube in abdominal cavity in the end of operation. Table 1 shows 186 patients’ characteristics.

**Table 1:** Clinical characteristics of 186 cases with appendectomy

Clinical characters	Number of cases (proportion, %)
Gender, male/female	89 (47.8%)/ 97 (52.2%)
Age, < 40 / ≥ 40 years	101 (54.3%)/ 85 (45.7%)
Operation, LA/OA	155 (83.3%)/ 31 (16.7%)
First onset, yes/no	118 (63.4%)/ 68 (36.6%)
Time interval from the onset to operation, days	6 no record
≤ 1	64 (34.4%)
1 < and ≤ 2	58 (31.2%)
2 < and ≤ 3	22 (11.8%)
> 3	36 (19.4%)
Time interval from initial onset to operation of recurrent patients, months	5 no record
≤ 3	9 (14.3%)
3 < and ≤ 12	25 (39.7%)
> 12	29 (46.0%)
Degree of intraoperative adhesions mild/middle/severe	92 (49.5%)/ 34 (18.3%)/ 60 (32.2%)
Intraoperative suppuration, yes/no	103 (55.4%)/ 83 (44.6%)
Intraoperative appendiceal perforation, yes/no	27 (14.5%)/ 159 (85.5%)
Operative time, mins	76 (20, 190)
Preoperative leukocyte count, × 10^9^/L	11.4 (2.1, 24.3)
Preoperative neutrophil percentage	74.6% (30.5%, 95.8%)
Preoperative US revealed periappendiceal fluid, yes/no	4 no record 35 (18.8%)/ 147 (79%)
Drainage tube, yes/no	115 (61.8%)/ 71 (38.2)
Pathology results, suppurative/ non-suppurative appendicitis	108 (58.1%)/ 78 (41.9%)

*LA* laparoscopic appendectomy, *OA* open appendectomy, *US* ultrasonography

Table 2 shows the intraoperative details and early postoperative outcomes of the three groups with different degrees of adhesions. Contrast to the moderate group (MoG) and the mild group (MiG), the severe degree of adhesions group (SG) has a longer operation time(mean: 98.6 vs. 71.5 vs. 62.8 min, *P* < 0.001), longer postoperative hospital stay (mean: 5.7 vs. 4.3 vs. 3.7 days, *P* = 0.002), higher intraoperative suppuration rate (66.7 vs. 64.7 vs. 44.6%, *P* = 0.013), higher intraoperative perforation rate(26.7 vs. 26.5 vs. 2.2%, *P* < 0.001), a greater likelihood of conversion to open (25.9 vs. 0 vs. 0%, *P* < 0.001), and more postoperative complications (10.0 vs. 8.8 vs. 1.1%, *P* = 0.036). All these differences between three groups are significant.

**Table 2:** Comparison of clinical characteristics of patients with different degrees of adhesions during appendectomy

	Degree of intraoperative adhesions			H /χ^2^ value	*P* value
	Mild	Middle	Severe		
Inpatient days, days	3.7 (1, 10)	4.3 (1, 10)	5.7 (1, 43)	12.136	0.002*
Intraoperative suppuration					
Yes	41	22	40	8.644	0.013*
No	51	12	20		
Intraoperative appendiceal perforation					
Yes	2	9	16	25.181	< 0.001*
No	90	25	44		
Operative time, mins	62.8 (20, 130)	71.5 (30, 130)	98.6 (35, 190)	32.797	< 0.001*
LA conversion to OA					
Yes	0	0	14	27.764	< 0.001*
No	75	26	40		
Extended resection					
Yes	1	0	3	2.590	0.310
No	91	34	57		
Complications					
Yes	1	3	6	7.283	0.015*
No	91	31	54		

*LA* laparoscopic appendectomy, *OA* open appendectomy, *OR* odds ratio, *CI* confidence interval

*P value is statistically significant

### Multivariate analysis

Table 3 shows a comparison of preoperative factors among three groups of patients (MoG, MiG and SG) with different degrees of adhesions during appendectomy. The gender and age of three groups were not significantly different (*P* = 0.475, *P* = 0.063 respectively). Time interval from symptom onset to operation of three groups were not significantly different (*P* = 0.361). The severe degree of adhesions group (SG) has a higher neutrophil percentage than other groups (MoG and MiG) (mean: 78.2 vs. 78 vs. 71%, *P* = 0.015). However, preoperative leukocyte count of three groups was not significantly different (*P* = 0.106). The severe degree of adhesions group (SG) has a higher rate of preoperative US revealed periappendiceal fluid than other groups (MoG and MiG) (28.8 vs. 23.5 vs. 11.2%, *P* = 0.023). Multivariable logistic regression analysis showed that the recurrent appendicitis (OR 95% CI 0.119, 1.589, *P* = 0.023) and high neutrophil percentage (OR 95% CI 0.014, 0.079, *P* = 0.005) were independently associated with the degree of appendiceal adhesions.

**Table 3:** Risk factors of adhesions during appendectomy identified by univariate and multivariate logistic regression analysis

	Univariate analysis					Multivariate analysis	
	Degree of intraoperative adhesions			H/χ^2^ value	*P* value	OR (95%CI)	*P* value
	Mild	Middle	Severe				
Gender							
Male	40	17	32	1.491	0.475	− 0.249, 0.956	0.250
Female	52	17	28			Reference	
Age, years	37.5 (14 ~ 78)	42.9 (17 ~ 81)	42.9 (14 ~ 78)	5.526	0.063	− 0.005, 0.032	0.151
First onset							
No	35	10	23	0.918	0.632	0.119, 1.589	0.023*
Yes	57	24	37			Reference	
Time interval from on-set to operation, days							
≤ 1	34	8	22	6.581	0.361	− 1.063, 0.879	0.853
1 < and ≤ 2	24	15	19			− 0.717, 1.193	0.626
2 < and ≤ 3	8	5	9			− 0.664, 1.618	0.412
> 3	21	6	9			Reference	
Preoperative leukocyte count, × 10^9^/L	10.6 (2.1 ~ 22.5)	13.1 (3.5 ~ 24.3)	11.7 (2.7 ~ 23.6)	4.487	0.106	− 0.090, 0.062	0.717
Preoperative neutrophil percentage, %	71 (30.5 ~ 95.8)	78 (45.9 ~ 94.3)	78.2 (45.6 ~ 93.6)	8.405	0.015*	0.014, 0.079	0.005*
Preoperative US revealed periappendiceal fluid							
No	79	26	42	7.555	0.023*	− 1.389, 0.168	0.124
Yes	10	8	17			Reference	

*OR* odds ratio, *CI* confidence interval, *US* ultrasonography

*P value is statistically significant

Table 4 shows a comparison of preoperative factors among three groups of patients (MoG, MiG and SG) with different degrees of adhesions in the recurrent appendicitis. Time interval from initial symptom onset to operation of three groups were not significantly different (*P* = 0.778). The gender, age, preoperative leukocyte count, preoperative US revealed periappendiceal fluid in three groups were all not significantly different (*P* = 0.361, *P* = 0.915, *P* = 0.488, *P* = 0.198 respectively). Although the severe degree of adhesions group (SG) has a higher neutrophil percentage than other groups (MoG and MiG) (mean: 73.7 vs. 61.9 vs. 63.7%, *P* = 0.010), multivariable logistic regression analysis showed that none of above were significant risk factors.

**Table 4:** Risk factors of adhesions during appendectomy in recurrent appendicitis identified by univariate and multivariate logistic regression analysis

	Univariate analysis					Multivariate analysis	
	Degree of intraoperative adhesions			H/χ^2^ value	*P* value	OR (95%CI)	*P* value
	Mild	Middle	Severe				
Gender							
Male	14	5	6	2.03	0.361	− 1.794, 0.597	0.327
Female	21	5	17			Reference	
Age, years	39.2 (17 ~ 67)	41.2 (17 ~ 73)	39.5 (22 ~ 60)	0.178	0.915	− 0.039, 0.033	0.853
Time interval from initial onset to operation of re-current patients, months							
≤ 3	4	1	4	1.940	0.783	− 0.986, 2.486	0.397
3 < and ≤ 12	15	4	6			− 1.316, 1.050	0.826
> 12	14	4	11			Reference	
Preoperative leukocyte count, × 10^9^/L	7.6 (2.1 ~ 17.1)	8.7 (3.5 ~ 18.6)	9.4 (2.7 ~ 19.1)	1.436	0.488	− 0.179, 0.168	0.952
Preoperative neutrophil percentage, %	61.9 (30.5 ~ 90.6)	63.7 (45.9 ~ 84.5)	73.7 (45.6 ~ 92.1)	9.174	0.010	0.005, 0.103	0.07
Preoperative US revealed periappendiceal fluid							
No	33	10	20	2.412	0.355	− 3.835, 1.309	0.336
Yes	1	0	3			Reference	

*OR* odds ratio, *CI* confidence interval, *US* ultrasonography

*P value is statistically significant

Table 5 shows a comparison of preoperative factors between suppurative and non-suppurative patients during appendectomy. The gender between two groups was not significantly different (*P* = 0.164). The age between the suppurative group and non-suppurative group was significantly different (mean: 42.4 vs. 37.5, *P* = 0.040). The proportion of the first onset between two groups was significantly different (*P* < 0.001). Time interval from symptom onset to operation of two groups was significantly different (*P* < 0.001). The preoperative leukocyte count and neutrophil percentage between the suppurative group and non-suppurative group were significantly different (mean: 13.8 vs. 8.4 × 10^9^/L, *P* < 0.001 and 82.5 vs. 64.7%, *P* < 0.001). The proportion of preoperative US revealed periappendiceal fluid between two groups was significantly different (31.0 vs. 4.9%, *P* < 0.001). Multivariable logistic regression analysis showed that only the preoperative US revealed periappendiceal fluid (OR 0.138, 95% CI 0.034, 0.561, *P* = 0.006) and high neutrophil percentage (OR 1.109, 95% CI 1.051, 1.169, *P* < 0.001) were independently associated appendix suppuration.

**Table 5:** Risk factors for appendix suppuration identified by univariate and multivariate logistic regression analysis

	Univariate analysis				Multivariate analysis	
	Non-suppurative	Suppurative	Z/χ^2^ value	*P* value	OR (95%CI)	*P* value
Gender						
Male	35	54	1.938	0.164	0.878 (0.363, 2.119)	0.772
Female	48	49			Reference	
Age, years	37.5 (16 ~ 73)	42.4 (14 ~ 81)	− 2.055	0.040*	1.025 (0.995, 1.056)	0.099
First onset						
No	48	20	29.242	< 0.001*	0.591 (0.223, 1.567)	0.290
Yes	35	83			Reference	
Time interval from on-set to operation, days						
≤ 1	21	43	29.967	< 0.001*	1.820 (0.355, 9.326)	0.472
1 < and ≤ 2	18	40			2.614 (0.525, 13.012)	0.241
2 < and ≤ 3	9	13			1.339 (0.200, 8.971)	0.764
> 3	30	6			Reference	
Preoperative leukocyte count, × 10^9^/L	8.4 (2.1 ~ 18.3)	13.8 (2.7 ~ 24.3)	− 6.734	< 0.001*	1.109 (0.975, 1.262)	0.116
Preoperative neutrophil percentage, %	64.7 (30.5 ~ 92.8)	82.5 (45.6 ~ 95.8)	− 8.129	< 0.001*	1.109 (1.051, 1.169)	< 0.001*
Preoperative US revealed periappendiceal fluid						
No	78	69	–	< 0.001*	0.138 (0.034, 0.561)	0.006*
Yes	4	31			Reference	

*OR* odds ratio, *CI* confidence interval, *US* ultrasonography

*P value is statistically significant

Table 6 shows a comparison of preoperative factors between perforated and unperforated patients during appendectomy. The gender and age between two groups were not significantly different (*P* = 0.199, *P* = 949 respectively). The proportion of the first onset, time interval from symptom onset to operation, the preoperative US revealed periappendiceal fluid between two groups were significantly different (*P* < 0.001, *P* = 0.020, *P* = 0.001 respectively). The preoperative leukocyte count and neutrophil percentage between the perforated group and unperforated group were significantly different (mean: 14.8 vs. 10.8 × 10^9^/L, *P* = 0.001 and 83.6 vs. 73.1%, *P* < 0.001). Multivariable logistic regression analysis showed that only high neutrophil percentage (OR 1.074, 95% CI 1.004, 1.150, *P* = 0.038) were independently associated appendix perforation.

**Table 6:** Risk factors for appendix perforation identified by univariate and multivariate logistic regression analysis

	Univariate analysis				Multivariate analysis	
	Unperforated	perforated	Z/χ^2^ value	*P* value	OR (95%CI)	*P* value
Gender						
Male	73	16	1.648	0.199	1.224 (0.447, 3.355)	0.694
Female	86	11			Reference	
Age, years	40.1 (14 ~ 81)	41.0 (17 ~ 78)	− 0.064	0.949	0.995 (0.966, 1.025)	0.752
First onset						
No	67	1	–	<	0.166 (0.020, 1.419)	0.101
Yes	92	26		0.001*	Reference	
Time interval from on-set to operation, days						
≤ 1	58	6	9.443	0.020*	0.210 (0.032, 1.379)	0.104
1 < and ≤ 2	48	10			0.475 (0.081, 2.789)	0.410
2 < and ≤ 3	14	8			1.584 (0.236, 10.637)	0.636
> 3	33	3			Reference	
Preoperative leukocyte count, × 10^9^/L	10.8 (2.1 ~ 24.3)	14.8 (2.7 ~ 22.8)	− 3.448	0.001*	1.074 (0.960, 1.201)	0.214
Preoperative neutrophil percentage, %	73.1 (30.5 ~ 95.8)	83.6 (60.3 ~ 94.1)	− 3.612	< 0.001*	1.074 (1.004, 1.150)	0.038*
Preoperative US revealed periappendiceal fluid						
No	132	15	10.400	0.001*	0.594 (0.202, 1.749)	0.344
Yes	24	11			Reference	

*OR* odds ratio, *CI* confidence interval, *US* ultrasonography

*P value is statistically significant

## Discussion

Acute appendicitis (AA) is the most common surgical emergency, but establishing the diagnosis of acute appendicitis based on clinical presentation and physical examination is still challenging. Several clinical scoring systems have been developed for early diagnosis of AA. The most popular for use in adult and children was the Alvarado score [15]. Two other systems such as the AIR score and the AAS score were also used currently, and they could decrease negative appendectomy rates in low-risk groups and reduce the need for imaging studies and hospital admissions in both low and intermediate risk groups [16–19]. All the cases in this study were definitively diagnosed with appendicitis before they were admitted to the hospital. We use the Alvarado score assess appendicitis in patients with abdominal pain in the emergency department. When the score was greater than 4, to further clarify the diagnosis, the patient was advised to have an US examination of appendix or plain CT scan of the lower abdomen with negative US findings. Patients established appendiceal abscess were treated with antibiotics and percutaneous drainage of abscess if needed. Others established simple or complex appendicitis were advised of surgical removal of the appendix.

Although urgent appendicectomy is still the recommended treatment for acute uncomplicated appendicitis, antibiotics have been proposed as a single treatment for uncomplicated appendicitis with controversy [4, 20, 21]. A meta-analysis of appendectomy vs. antibiotic treatment showed that although antibiotic treatment alone can be successful, a failure rate at 1 year was around 25–30% with need for readmission or surgery [22]. It was suggested that an antibiotics-first strategy may be considered in those who have strong preferences for avoiding an operation or who have contraindications to surgery [23]. In the Shandong Provincial Hospital affiliated to Shandong First Medical University between January 2018 and December 2019, there were 186 patients (86.1%) treated with urgent appendicectomies eligible, and there were 30 patients (13.9%) treated with antibiotics and other managements. In the operating patients of this study, the first onset of appendicitis accounted for 63.4% and recurrent appendicitis for 36.6%. Whether to have appendectomy were consistent with the patients’ decisions according to the doctor’s advice, and there was no negative appendicectomy. All the appendectomies were successful and no death was reported, with a laparoscopic appendectomy rate of 83.3% and open appendectomy rate of 16.7%. Our study supports that urgent appendicectomy using LA or OA in patients which were definitely diagnosed by relevant symptoms, signs, laboratory and imaging results were safe and effective.

In this study, the majority of patients with acute appendicitis can be successfully managed with laparoscopy, however, the operation time ranged from 20 to 190 min, 14 (9.0%) of LA converted to OA, 4 patients (2.2%) performed extended ileocecal resection instead of appendicectomy, postoperative complications occurred in 10 patients (5.4%). Previous studies showed that the most common reasons for conversion from laparoscopic to open appendectomy are severe inflammatory adhesions, a pre-operative diagnosis of complicated appendicitis(perforated or gangrenous appendicitis), presence of peri-appendicular abscess and diffuse peritonitis, because of obscuring the anatomy or resulting in friability or perforation [10, 11, 13]. And we found that the main reason of conversion-to-open and extended resection was adhesions as recorded in the operation notes. Peritoneal adhesions or intra-abdominal adhesions mean the state of close connection caused by the fibrous tissue between abdominal organs and peritoneum. They are often caused by inflammation, injury, ischemia and other reasons, and make operations difficult. All 14 patients of conversion-to-open had intraoperative severe degree of adhesions, laparoscopic appendectomy could be hardly completed in such cases because appendix and other organs around were badly attached and it was easy to damage the intestine or other organs without the hand touch feedback. We also found that severe adhesions were contributed to longer operation time, longer postoperative hospital stay, higher intraoperative suppuration rate, higher intraoperative perforation rate, and more postoperative complications. Therefore, it is necessary to find out risk factors of severe periappendiceal adhesions and give appropriate treatment.

The experienced veteran surgeons of our hospital believed that appendicitis lasting more than 3 days was not appropriate for surgical treatment because of severe abdominal adhesions. Most of these patients were supported with antibiosis and percutaneous drainage of abscess if needed. However, outcomes in relation to timing of surgery have been controversial. Some studies show that a longer length of preoperative stay significantly increased the risk of complications and mortality, also caused extensive resection for acute appendicitis [24–27]. And other studies show that short delays of less than 24 h before appendicectomy were not associated with increased rates of complex pathology in selected patients, but symptomatic time > 48 h were independently associated with complications [28, 29]. To make clear whether a longer length of preoperative stay was contributed to more adhesions, we divided the time interval from symptom onset to operation into four groups: ≤ 1 day, > 1 day and ≤ 2 days, > 2 days and ≤ 3 days, > 3 days, and took whether the first onset of appendicitis into account. The difference of intraoperative adhesions in each group was compared. Univariate analysis and multivariate logistic regression analysis showed no difference between each group of the time interval from symptom onset to operation. The risk factors influencing periappendiceal adhesions were recurrent appendicitis and high neutrophil percentage before operation. This suggests that chronic inflammation is a possible cause of periappendiceal adhesions, and those recurrent appendicitis with high neutrophil percentage had higher risk of adhesions. Furthermore, to investigate whether the time interval from initial symptom onset to operation was related to adhesions in recurrent appendicitis, we divided it into three groups: ≤ 3 months, > 3 months and ≤ 12 months, > 12 months. Univariate analysis showed no difference between each group of different time interval. Multivariate logistic regression analysis showed no preoperative factors of adhesions in recurrent appendicitis were significant.

It was recommended that POCUS (point-of-care US) was the most appropriate first-line diagnostic tool in both adults and children, if an imaging investigation was indicated based on clinical assessment [17]. Overall sensitivity and specificity of US is 76% and 95% and for CT is 99% and 84% respectively [30], and appendicitis could be effectively diagnosed with them. However, the usefulness of CT for determining perforation or adhesions in AA is limited [31]. In our hospital, We use US more than CT for diagnosing appendicitis because of no radiation, cheaper and more convenient. And we tried to find more evidence that US predicted appendiceal suppuration, perforation or adhesions. As the results showed, preoperative US with periappendiceal fluid was a risk factor of appendix suppuration, but not of appendix perforation and adhesions. Multivariate logistic regression analysis showed that an increased percentage of neutrophils was the only risk factor of appendix perforation, and it was also a risk factor of appendix suppuration.

In this study, we found that recurrent appendicitis and increased preoperative neutrophil percentage are risk factors for intraoperative periappendiceal adhesions, and adhesions can lead to prolonged operation time and hospital stay, more often accompanied by suppuration and perforation, resulting in more postoperative complications. Therefore, for those patients with severe adhesions, immediate appendectomy should be carefully evaluated. For some patients, antibiotics should be given first, and the appendix can be removed at an elective time 3 months later when periappendiceal adhesions was reduced, so as to reduce the surgical risks caused by adhesions. Nevertheless, the present study has some limitations. It is a retrospective analysis and a single-center study; therefore, it has some inherent biases. The judgement of the appendiceal degree of adhesions may be objective and was based on the operative surgeons. The US examinations were not performed by a single person. The preoperative risk factors studied were not comprehensive, CT and CRP were not routinely examined and included in the study.

## Conclusions

In cases of appendicitis, we demonstrated that recurrent appendicitis and preoperative high neutrophil percentage were risk factors of periappendiceal adhesions; preoperative US revealed periappendiceal fluid and high neutrophil percentage were risk factors of appendix suppuration; and a high preoperative neutrophil percentage was a risk factor of appendix perforation. In this study, no effect of operation timing on appendiceal adhesions, suppuration and perforation was found.

## Data Availability

The data used and analyzed during the current study are available from the corresponding author on reasonable request.
